# Fear-themed digital media exposure and sleep regulatory sensitivity in school-aged children: preliminary observations toward a developmental PhenoSleep construct

**DOI:** 10.3389/fneur.2026.1816004

**Published:** 2026-05-22

**Authors:** Martina Gnazzo, Giuditta Bargiacchi, Eva Germanò, Agata Maltese, Lucia Parisi, Laura Firrigno, Michele Roccella, Giulia Spoto, Gabriella Di Rosa, Lidia Scifo, Beatrice Gallai, Davide Testa, Annamaria Maddalena Terracciano, Marco Carotenuto

**Affiliations:** 1 Department of Biomedical, Metabolic and Neural Sciences, University of Modena and Reggio Emilia, Modena, Italy; 2 Sleep Lab for Developmental Age, Clinic of Child and Adolescent Neuropsychiatry, Department of Mental and Physical Health and Preventive Medicine, Child and Adolescent Neuropsychiatry Clinic, University of Campania “Luigi Vanvitelli”, Naples, Italy; 3 The PRINCE Network (Pediatric Regulatory and Integrative Neurodevelopmental Circuits and Endophenotypes), Naples, Italy; 4 Child and Adolescent Neuropsychiatry Unit, Department of Human and Evolutionary Pathology “Gaetano Barresi”, University of Messina, Messina, Italy; 5 Department of Psychology, Educational Science and Human Movement, University of Palermo, Palermo, Italy; 6 Unit of Child Neurology and Psychiatry, Department of Biomedical Sciences, Dental Sciences and Morpho-Functional Imaging, University of Messina, Messina, Italy; 7 Department of Law, Economics and Communication (Palermo), LUMSA University of Rome, Palermo, Italy; 8 Department of Surgical and Biomedical Sciences, University of Perugia, Perugia, Italy; 9 Casa di Cura Santa Maria del Pozzo Hospital, Somma Vesuviana, Naples, Italy

**Keywords:** behavioral assessment, CBCL, children, Huggy Wuggy, media effects, SDSC

## Abstract

**Introduction:**

Fear-themed digital media exposure is increasingly common in school-aged children, yet its developmental implications remain insufficiently understood. Rather than conceptualizing frightening content as inherently pathogenic, emotionally intense media may act as a mild arousal stimulus within a neurodevelopmental system characterized by heightened limbic reactivity and still-maturing regulatory control. The present study examined whether fear-themed exposure is associated with dimensional variability in sleep regulatory sensitivity, consistent with a tentative developmental PhenoSleep construct.

**Methods:**

This cross-sectional study included 132 children aged 5–11 years (mean age = 8.69 years, SD = 2.01), divided into an exposed group (*n* = 66) and a non-exposed control group (n = 66). Engagement with fear-themed content (Huggy Wuggy) was assessed using an *ad hoc* exposure questionnaire (TOT_HW). Emotional-behavioral functioning was measured with the Child Behavior Checklist (CBCL), and sleep regulation with the Sleep Disturbance Scale for Children (SDSC). Independent-sample *t*-tests and Pearson's correlations were performed.

**Results:**

Compared to controls, exposed children showed higher CBCL Total Problems and SDSC Total Scores. Domain-specific differences were modest and not uniformly distributed across subscales. Within the exposed group, engagement intensity showed small positive associations with selected social, cognitive, and sleep-related measures, consistent with dimensional variability rather than overt dysfunction.

**Conclusion:**

The findings do not support a deterministic psychopathological effect of fear-themed digital exposure. Instead, they are compatible with the interpretation that emotionally salient media may function as a mild environmental probe revealing interindividual differences in sleep regulatory sensitivity. We tentatively introduce the PhenoSleep construct to describe developmental variability in sleep continuity and arousal modulation in response to emotionally intense stimuli. Longitudinal and physiologically informed studies are required to further examine and refine this developmental regulatory model.

## 1 Introduction

Throughout human history, fear-inducing narratives have represented a stable component of cultural transmission, from mythological archetypes to fairy tales and contemporary digital media (1, 2). Rather than being inherently pathological, exposure to emotionally intense content has traditionally functioned as a symbolic space for processing threat and uncertainty within controlled environments.

In childhood, emotional processing is characterized by heightened limbic reactivity within the context of still-maturing prefrontal regulatory systems. The amygdala, locus coeruleus, and noradrenergic pathways demonstrate high responsivity to threat-related stimuli, while cortical inhibitory modulation remains developmentally incomplete. This neurodevelopmental configuration may result in prolonged arousal responses and increased sensitivity to emotionally salient environmental inputs (1–5).

Fear-themed content consumed in contexts perceived as safe may paradoxically combine limbic activation with reward system engagement. Dopaminergic reinforcement mechanisms may coexist with autonomic arousal, generating a state of emotionally charged but controlled stimulation. Within this framework, such content may not necessarily induce psychopathology but may instead act as a mild arousal probe capable of revealing interindividual differences in regulatory sensitivity (3).

Sleep regulation represents a particularly sensitive system within this bidirectional arousal–regulation interplay. Given the reciprocal relationship between emotional activation and sleep continuity, repeated exposure to fear-themed digital stimuli may not produce overt behavioral dysregulation but could be associated with subtle variability in sleep continuity, pre-sleep cognitive activation, and nocturnal arousal modulation (4–8).

In previous observational work, imitation of violent streaming content was associated with subtle somatic complaints in children, without evidence of broad behavioral dysregulation (9). The present study extends this line of inquiry by shifting the focus from somatic expression to sleep regulatory processes.

In light of these considerations, we tentatively introduce the concept of a developmental sleep regulatory phenotype—here referred to as the PhenoSleep construct—characterized by dimensional variability in sleep continuity and arousal modulation in response to emotionally salient environmental stimuli.

We hypothesized that fear-themed digital media exposure would be associated with dimensional variations in sleep regulatory sensitivity, consistent with the preliminary contours of the PhenoSleep construct. Rather than conceptualizing fear-themed exposure as a direct causal factor for sleep pathology, the present study aims to explore whether such exposure may function as a mild arousal probe capable of revealing individual differences in developmental sleep regulation.

## 2 Methods

### 2.1 Ethical statement

This cross-sectional observational study was carried out in multiple primary schools in Palermo, Italy, after receiving approval from the Ethics Committee of the University of Palermo (approval code no. 76317-2024; 30 May 2024). Data were collected over one academic term, following ethical clearance and in close collaboration with school principals and teaching personnel.

### 2.2 Participants

A total of 132 children (66 males and 66 females), aged 5–11 years, were included in the study. Participants were divided into a Huggy Wuggy exposure group (HW; n = 66; mean age = 8.75 years, SD = 2.14) and a matched control group (*n* = 66; mean age = 8.63 years, SD = 1.88).

### 2.3 Instruments

*Ad Hoc Huggy Wuggy questionnaire:*

This 15-item *ad hoc* questionnaire assessed the degree of knowledge and engagement with Huggy Wuggy content. Scores ranged from 0 to 30, with higher scores indicating greater engagement. The questionnaire was designed to capture heterogeneous aspects of media exposure rather than to operationalize a unidimensional construct; therefore, psychometric reliability indices were not computed. The total score (TOT_HW) was calculated as the sum of item responses, yielding a composite index (range 0–30). Although formal psychometric validation was not performed, items were designed to cover multiple dimensions of exposure (frequency, emotional valence, and social engagement), supporting preliminary content validity. The questionnaire items are reported in Supplementary Material 1.

The total score (TOT_HW; range 0–30) reflects a composite index of familiarity, emotional response, and behavioral engagement.

Given the multidimensional and exploratory nature of the instrument, internal consistency was not computed.

*Child behavior checklist (CBCL):*

The Italian validated version of the Child Behavior Checklist (CBCL) was administered to caregivers. The CBCL is a well-established parent-report instrument designed to assess emotional and behavioral functioning across multiple domains, including Withdrawn/Depressed behavior, Somatic Complaints, Anxiety/Depression, Social Problems, Thought Problems, Attention Problems, Rule-Breaking Behavior, Aggressive Behavior, as well as broader Internalizing, Externalizing, and Total Problems scales. The instrument provides both syndrome scales targeting specific areas of difficulty and composite scales offering a more global behavioral profile. Raw scores are converted into age- and gender-normed T-scores, with higher values indicating greater symptom severity. For syndrome scales, *T*-scores ≥ 70 fall within the clinical range, scores between 65 and 69 indicate a borderline range, and scores ≤ 64 are considered non-clinical. For composite scales and the Total Problems scale, T-scores ≥ 64 are classified as clinical, 60–63 as borderline, and ≤ 59 as non-clinical. These standardized thresholds, derived from normative samples, support consistent interpretation in both clinical and research settings (10).

*Sleep disturbance scale for children (SDSC)*

This scale is worldwide used in order to assess the sleep habits and disturbances in developmental age across six domains, including Disorders of Initiating and Maintaining Sleep (DIMS), Sleep-Related Breathing Disorders (SRBD), Disorders of Arousal (DA), Disorders of Wake-Sleep Transition (DWST), Disorders of Excessive Somnolence (DOES), and Sleep Hyperhidrosis (SHY). The internal consistency (Cronbach's alpha) was 0.71 (11). The SDSC scale is a caregiver-reported measure designed to assess sleep-related disturbances; it does not capture objective parameters of sleep architecture. Despite this limitation, the instrument is extensively employed in school-aged populations and has demonstrated adequate psychometric properties in Italian samples. Given that the SHY subscale does not align with a formal sleep disorder category as defined by the International Classification of Sleep Disorders (ICSD), it was not considered in the primary analytical model. Within the present study, the SDSC was used as a dimensional proxy of sleep continuity and arousal-related variability, consistent with the tentative conceptualization of the PhenoSleep developmental phenotype construct.

### 2.4 Procedure

Data were collected during the academic year through paper-based questionnaires completed by caregivers. Group allocation was determined based on parental reports of their child's exposure to Huggy Wuggy–related digital content. The CBCL and SDSC were administered alongside the *ad hoc* exposure questionnaire.

The questionnaire was designed to capture multiple dimensions of exposure, including familiarity, emotional valence (e.g., fear vs. enjoyment), frequency of engagement, and social sharing behaviors, supporting its content relevance despite the lack of formal validation.

### 2.5 Data analysis

Descriptive statistics were computed for all variables and are presented in Table 1. The HW and control groups did not differ significantly in age (*p* = 0.73). Compared to controls, the HW group exhibited significantly higher scores on the CBCL Total Problems scale (*p* < 0.001) and the SDSC Total Score (*p* < 0.001), reflecting greater dimensional variability in emotional–behavioral functioning and sleep continuity.

**Table 1:** Sociodemographic and clinical characteristics of the sample.

Variable	HW (*n* = 66)	Controls (*n* = 66)	*p-value*
Age—M (SD)	8.75 (2.14)	8.63 (1.88)	0.73
CBCL_TOT—M (SD)	72.03 (3.21)	46.39 (2.82)	**<0.001**
SDSC_TOT—M (SD)	61.76 (4.24)	58.98 (2.86)	**<0.001**

Group differences were assessed using independent-sample t-tests; corresponding t values and degrees of freedom are reported in the Results section. P-value < 0.05 was considered statistically significant. Bold values indicate statistically significant differences.

Group comparisons for continuous variables were performed using independent-sample *t*-tests after checking for normality.

## 3 Results

### 3.1 Group characteristics

Each group (HW and controls) included 66 participants, with balanced mean ages and gender distributions. No significant difference in age was found between the groups (*p* = 0.73). Compared to controls, the HW group exhibited significantly higher scores on the CBCL Total Problems scale (*t* = 48.8, df = 130, *p* < 0.001) and the SDSC Total Score (*t* = 4.42, df = 130, *p* < 0.001), indicating greater psychological and sleep-related challenges.

### 3.2 Group comparisons

Independent-sample *t*-tests showed no statistically significant differences between groups in CBCL Thought Problems (*t* = 1.78, df = 130, *p* =0.078) and Social Problems (*t* = 1.70, df = 130, *p* = 0.092), although both domains showed a trend toward higher scores in the HW group.

These findings should be interpreted as non-significant exploratory patterns.

**Figure 1:**
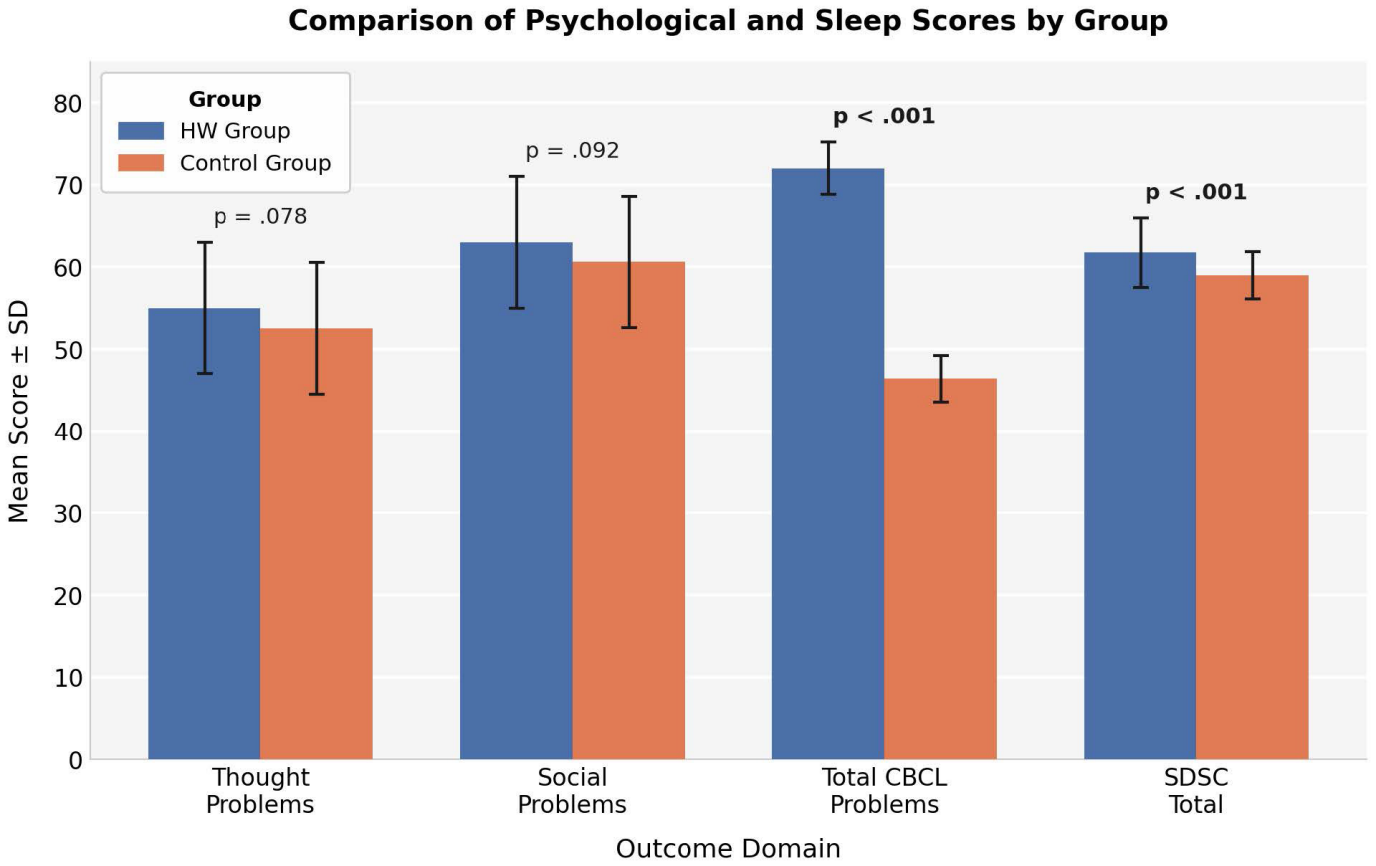
Comparison of mean scores (± standard deviation) between children exposed to Huggy Wuggy content (HW group) and the control group across CBCL total problems, SDSC total score, thought problems, and social problems. Independent-sample *t*-test results are reported as follows: CBCL total problems (*t* = 48.8, df = 130, *p* < 0.001), SDSC total score (*t* = 4.42, df = 130, *p* < 0.001), thought problems (*t* = 1.78, df = 130, *p* = 0.078), and social problems (*t* = 1.70, df = 130, *p* = 0.092). Error bars represent standard deviation.

These patterns are visually summarized in Figure 1, which shows the mean scores (± SD) across the main CBCL and SDSC domains for both groups.

### 3.3 Correlational analyses

**Figure 2:**
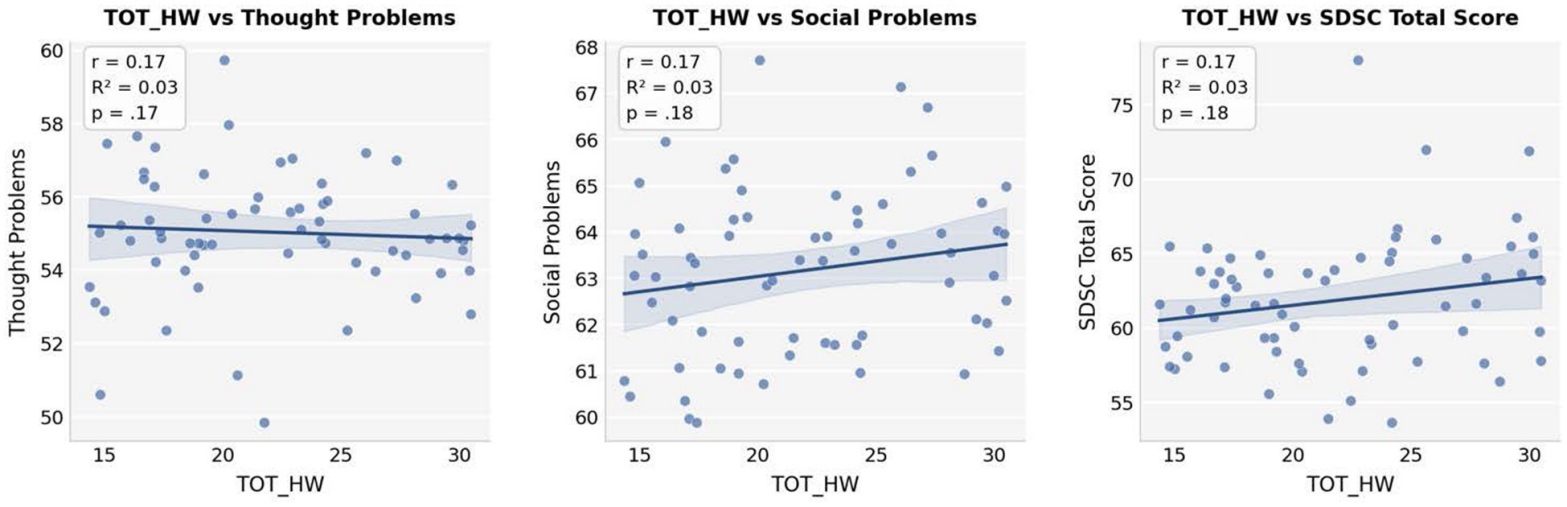
Scatterplots with regression lines illustrating the relationship between TOT_HW scores and outcome domains (thought problems, social problems, and SDSC total score). Pearson correlation coefficients (*r*), coefficients of determination (*R*^2^), and *p*-values are reported within each panel. No statistically significant associations were observed.

Within the HW group, Pearson correlations showed small to moderate positive relationships between TOT_HW and Social Problems (*r* = 0.17, *R*^2^ = 0.03, *p* = 0.18) and Thought Problems (*r* = 0.17, *R*^2^ = 0.03, *p* = 0.17), although these were not statistically significant. Other domains exhibited weak or negligible connections. To visually examine these relationships, scatterplots were reviewed (Figure 2). Although no correlation was confirmed as significant, small positive patterns were seen between TOT_HW and Thought Problems, Social Problems, and SDSC Total Score, indicating that greater engagement with Huggy Wuggy content might be associated with slight increases in cognitive and emotional symptoms.

## 4 Discussion

The present findings should be interpreted within a developmental regulatory framework rather than a strictly psychopathological model. Although children exposed to fear-themed digital content displayed higher total CBCL and SDSC scores, domain-specific impairments were not consistently robust. This pattern suggests that exposure may not directly induce clinically significant dysfunction, but may instead interact with underlying regulatory sensitivity during a developmental phase characterized by heightened limbic responsivity and still-maturing prefrontal modulation.

Within this perspective, fear-themed digital stimuli may function as mild arousal probes rather than pathogenic agents. Emotional activation triggered by frightening content is likely mediated by amygdala-driven threat detection systems and noradrenergic arousal pathways, which are developmentally more reactive in childhood. When such activation occurs in perceived safe environments, it may coexist with dopaminergic reward engagement, creating a state of controlled emotional stimulation. This dual activation may not necessarily translate into overt behavioral dysregulation but could reveal subtle variability in regulatory domains—particularly sleep.

Sleep represents one of the most sensitive regulatory systems to emotional arousal. Previous literature has consistently shown that emotionally stimulating or violent media exposure may negatively influence sleep onset, continuity, and overall quality (12, 13). However, much of this research has focused on pathological or clearly violent content and on overt sleep disturbances. In contrast, the present findings suggest a more dimensional pattern: exposed children exhibited higher total sleep disturbance scores, yet without a uniform elevation across all sleep domains. This variability aligns with the hypothesis that emotionally salient media may interact with individual differences in arousal modulation rather than producing uniform sleep pathology. A similar pattern emerged in emotional-behavioral domains. Although the HW group demonstrated higher overall CBCL total scores, subscale differences were modest, with marginal trends in Thought Problems and Social Problems. Prior research has shown that exposure to disturbing media content may influence children's cognitive processing, internal representations of threat, and interpretation of social cues (14, 15). However, the absence of strong domain-specific impairments in our sample suggests that fear-themed exposure may not act as a direct cause of behavioral dysregulation, but rather as a contextual stressor interacting with pre-existing regulatory traits.

Importantly, these findings extend previous observational evidence in which imitation of violent streaming content was associated with subtle somatic expressions of arousal without generalized psychopathology (9). In that study, exposure was linked to increased somatic complaints rather than broad behavioral disturbance. The current results suggest that sleep regulation may represent another sensitive domain through which emotionally intense media exposure becomes observable at a subclinical level.

In light of these patterns, we tentatively introduce the concept of a developmental sleep regulatory phenotype, referred to here as the PhenoSleep construct. This construct describes dimensional variability in sleep continuity and arousal modulation in response to emotionally salient environmental stimuli. Within this framework, fear-themed digital content does not function as a deterministic risk factor, but rather as a mild environmental probe capable of revealing individual differences in regulatory sensitivity. Some children may demonstrate stable sleep continuity despite exposure, whereas others may exhibit increased pre-sleep cognitive activation, nocturnal arousal, or subtle sleep fragmentation.

This interpretation is consistent with broader models of media effects suggesting that outcomes are moderated by developmental stage, temperament, family context, and media mediation strategies (16). The interaction between emotional activation and sleep regulation is inherently bidirectional; emotional arousal can disrupt sleep, and insufficient sleep can impair emotional regulation (12, 17). Therefore, repeated exposure to emotionally charged content may amplify existing vulnerabilities rather than generate pathology *de novo*.

The engagement measure was based on an *ad hoc* questionnaire lacking formal psychometric validation. Therefore, the TOT_HW score should be interpreted as an exploratory proxy of exposure rather than a standardized measure. Future studies should employ validated instruments or develop psychometrically robust tools to quantify fear-themed media engagement. Furthermore, the absence of physiological sleep measures (e.g., actigraphy, heart rate variability, and EEG markers of arousal modulation) constrains the interpretation of regulatory mechanisms. Future research should incorporate multimodal assessment strategies and longitudinal designs to determine whether the observed patterns represent stable developmental phenotypes or transient regulatory fluctuations. Despite these limitations, the present study contributes to a reframing of the discourse surrounding fear-themed digital media exposure in childhood. Rather than positioning frightening content exclusively as a risk factor, our findings suggest that such exposure may serve as a context in which regulatory sensitivity becomes observable. The proposed PhenoSleep construct offers a preliminary developmental model for understanding how emotionally salient environmental stimuli may interact with sleep continuity and arousal modulation during childhood. Several potential confounding variables were not systematically assessed, including baseline screen time, parental mediation strategies, bedtime routines, and individual differences in media access. These factors may influence both exposure to fear-themed content and sleep or behavioral outcomes. Additionally, reliance on caregiver reports introduces the possibility of reporting bias, particularly in parents more attentive to media-related concerns. Future studies should incorporate these variables to better disentangle exposure-related effects from broader environmental and familial influences.

Future research should integrate objective physiological measures of sleep and arousal regulation, such as actigraphy, heart rate variability, and EEG-based indices of arousal instability, to better characterize the neurobiological substrates underlying the proposed PhenoSleep framework.

## 5 Conclusion

In conclusion, the present findings suggest that exposure to fear-themed digital media may be associated with dimensional variations in emotional-behavioral functioning and sleep continuity in school-aged children. These differences do not appear to reflect uniform or clinically significant dysfunction, but rather patterns consistent with variability in developmental regulatory sensitivity. Within this perspective, fear-themed content may function less as a deterministic risk factor and more as an environmental context in which individual differences in sleep–emotion modulation become observable. The tentative introduction of the PhenoSleep construct offers a dimensional framework for understanding how emotionally salient stimuli may interact with sleep continuity during sensitive developmental phases. From a preventive standpoint, these findings underscore the importance of contextual mediation rather than simple content restriction. Media education initiatives aimed at parents, caregivers, and educators may support children in processing emotionally intense material within safe and reflective environments. Developmentally informed guidance—particularly regarding pre-sleep media exposure—may help preserve sleep continuity and promote adaptive emotional regulation.

These findings may also be aligned with broader evidence indicating that environmental exposures interact with neurodevelopmental vulnerability across multiple conditions, including neurodevelopmental syndromes and stress-related psychopathology, where external stimuli reveal underlying regulatory sensitivity rather than acting as deterministic causal factors (18, 19).

Future longitudinal and multimodal investigations incorporating objective sleep measures and physiological indices of arousal will be essential to determine whether the observed patterns represent stable developmental phenotypes or transient regulatory responses.

## Data Availability

The raw data supporting the conclusions of this article will be made available by the authors, without undue reservation.
