# Supplementary material for: Fear-themed digital media exposure and sleep regulatory sensitivity in school-aged children: preliminary observations toward a developmental PhenoSleep construct
Source: Front Neurol. 2026 May 22;17:1816004. doi: 10.3389/fneur.2026.1816004 (PMC13236553; doi:10.3389/fneur.2026.1816004)
Supplement: Supplementary file 1 [file Supplementary_file_1.docx]

**Supplementary Material 1. Questionnaire (Italian version)**

- **Sesso:** MASCHIO FEMMINA
- **QUANTI ANNI HAI?**

10

11

9

8

7

5

6


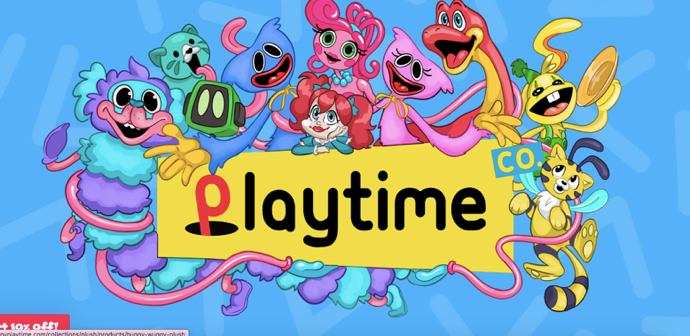


1. **Hai mai giocato con “Poppy Playtime?**

A: Si

B: No

1. **Quante volte ci giochi?**
2. Mai
3. A volte
4. Tutti i giorni
5. Più volte al giorno


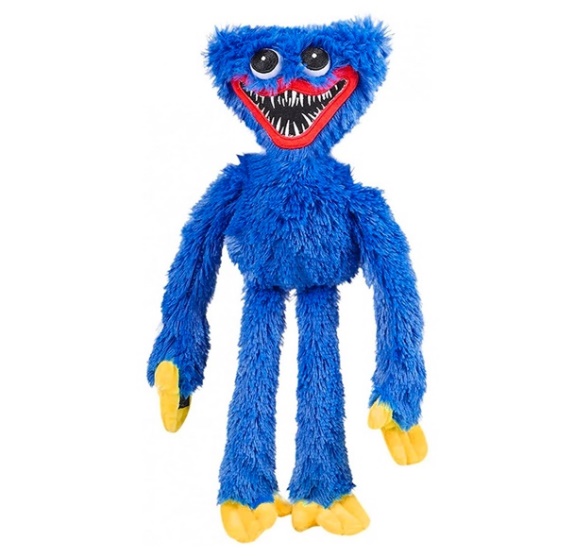


1. **Ti piace questo gioco?**
2. Si
3. No
4. **Conosci questo personaggio?**

A: Si

B: No

1. **Come si chiama?**
2. Huggy Wuggy
3. Muppet
4. Peppa Pig
5. Blu
6. **Come lo hai conosciuto?**
7. Me ne hanno parlato.
8. Ho visto un peluche.
9. Ho giocato con lui.
10. Ho visto dei video su YouTube.
11. **Cosa pensi di questo personaggio?**
12. Penso che sia bello, buono e divertente
13. Mi fa paura
14. È famoso, ma a me non piace
15. Mi piace e ci vorrei giocare
16. **Hai mai visto video su YouTube con Huggy Wuggy?**
17. Si
18. No
19. **Hai mai parlato di questo gioco con i tuoi amici?**
20. Si,
21. No
22. A Volte
23. **I tuoi genitori conosco Huggy Wuggy?**
24. Si
25. No
26. **Conosci altri pupazzi simili ad Huggy Wuggy?**
27. Si
28. No
29. **Conosci la canzone di Huggy Wuggy?**
30. Si
31. No
32. **Ti piacciono le sue canzoni?**
33. Si
34. No
35. **Hai mai visto per strada o in negozi di giocattoli il peluche di Huggy Wuggy?**
36. Si
37. No
38. **Hai mai comprato o ti hanno regalato il suo peluche?**
39. Si
40. No
